# Evaluation-oriented exploration of photo energy conversion systems: from fundamental optoelectronics and material screening to the combination with data science

**DOI:** 10.1038/s41428-020-00399-2

**Published:** 2020-08-28

**Authors:** Akinori Saeki

**Affiliations:** Department of Applied Chemistry, Graduate School of Engineering, Osaka University, Suita, 565-0871 Japan

**Keywords:** Photochemistry, Polymer chemistry, Physical chemistry

## Abstract

Light is a form of energy that can be converted to electric and chemical energies. Thus, organic photovoltaics (OPVs), perovskite solar cells (PSCs), photocatalysts, and photodetectors have evolved as scientific and commercial enterprises. However, the complex photochemical reactions and multicomponent materials involved in these systems have hampered rapid progress in their fundamental understanding and material design. This review showcases the evaluation-oriented exploration of photo energy conversion materials by using electrodeless time-resolved microwave conductivity (TRMC) and materials informatics (MI). TRMC with its unique options (excitation sources, environmental control, frequency modulation, etc.) provides not only accelerated experimental screening of OPV and PSC materials but also a versatile route toward shedding light on their charge carrier dynamics. Furthermore, MI powered by machine learning is shown to allow extremely high-throughput exploration in the large molecular space, which is compatible with experimental screening and combinatorial synthesis.

## Abstract (web summary)

This article reviews an evaluation-oriented exploration of photo energy conversion systems including organic photovoltaics, perovskite solar cells, photocatalysts, and photodetectors. A time-resolved spectroscopy using a gigahertz electromagnetic wave enables rapid screening of potential optoelectronics of organic/inorganic semiconductors and fast finding of their optimal film processing conditions. This approach is further empowered by machine learning that provides a high-throughput virtual screening in the large molecular space. The author discusses a perspective on this evaluation (from fundamental to application) and its effective combination with data science.

## Graphical abstract

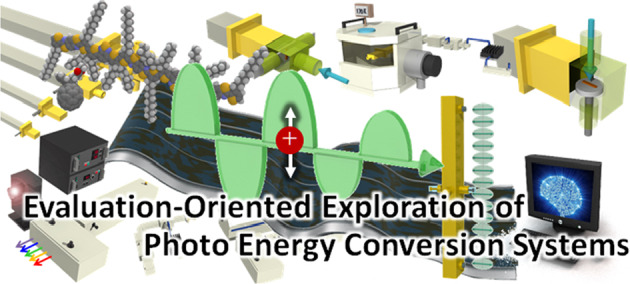

## Introduction

π-Electrons that mostly occupy the highest occupied molecular orbital (HOMO) and the lowest unoccupied molecular orbital (LUMO) of aromatic compounds are of particular interest because they exhibit intriguing responses to external stimuli such as photons, electric fields, and magnetic fields [1–3]. Electronic excitation by a photon with an energy greater than the HOMO–LUMO gap (bandgap, *E*_g_) generates an exciton (a pair of holes and electrons coupled with phonons), the energy of which can be converted to electricity when the exciton is dissociated into free charge carriers (hole and electron) that are separately transported to the respective electrodes [4–6]. However, the exciton binding energy of a π-conjugated material is much larger than the thermal energy at room temperature due to the small dielectric constant, whereas an inorganic semiconductor undergoes prompt charge separation owing to its large dielectric constant and small effective mass of charges [7, 8]. Accordingly, organic photovoltaics (OPVs) require a bulk heterojunction (BHJ) structure composed of a bicontinuous network of electron donor and acceptor materials (positive [p] and negative [n] semiconductors, respectively), which offers an energetic driving force for efficient charge separation at the enlarged interfacial area [9, 10].

**Fig. 1:**
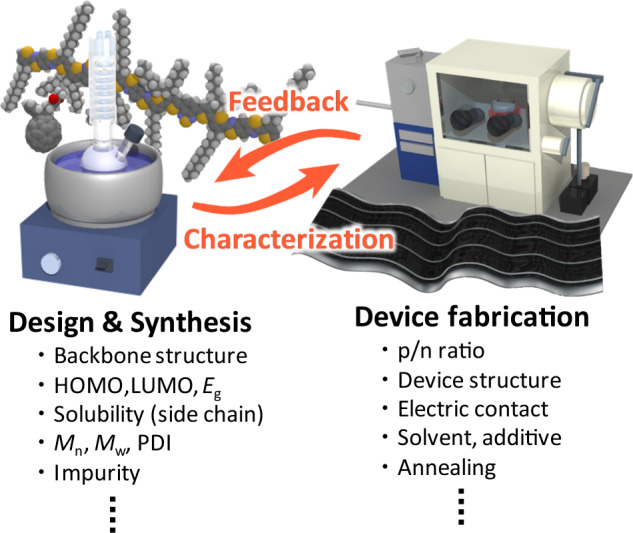
Schematic of OPV development consisting of (left panel) material design and synthesis and (right panel) device fabrication and characterization, which includes various factors to be considered as listed below the image

Since the power conversion efficiency (PCE) of a solar cell is defined by the product of the short-circuit current density (*J*_SC_), open-circuit voltage (*V*_OC_), and fill factor (FF) [PCE = *J*_SC_ × *V*_OC_ × FF (*P*_in_)^−1^, where *P*_in_ is the incident sunlight power density, typically 100 mW cm^−2^ of the pseudo-sunlight: air mass 1.5 global (AM1.5G)], maximizing all of these parameters is a direct way to improve PCE; however, *J*_SC_ and *V*_OC_ are inversely related because of the interplay among the bandgap excitation, sunlight spectrum, and electrochemical property. Shockley and Queisser calculated the PCE limit to be ~33% for a single p–n junction solar cell at *E*_g_ ~ 1.3 eV [11], while the optimal *E*_g_ shifts to a larger value when the energy loss becomes large like OPV due to its large exciton binding energy, nonradiative charge recombination, energy offset to ensure charge separation, etc. [12–15]. The design of OPV materials is therefore complicated, as shown in Fig. 1, where both the backbone structure, which predominantly determines its HOMO, LUMO, and *E*_g_, and the side chains, which are associated with solubility and BHJ morphology, have a great impact on the device performance. In the case of π-conjugated polymers, their molecular weights (the number-averaged molecular weight, *M*_n,_ and the weight-averaged molecular weight, *M*_w_) and polydispersity indices (PDIs) are closely related to the crystallinity and miscibility with an n-type semiconductor. Impurities such as residues of metal catalysts and target substituents for cross coupling polycondensation often act as charge traps, which should be removed as much as possible. Moreover, OPV device characterization requires tedious optimization of the p/n blend ratio, device structure, solvent, additive, and thermal annealing. After the PCE reaches a maximum value for each conjugated material, feedback from the results is applied to the molecular design, and incremental modification and synthesis are performed. This kind of research cycle requires considerable time and effort, which precludes the rapid development of OPVs.

Time-resolved spectroscopies using an electromagnetic wave (EMW) as the probe can directly measure the dynamics of transient species (charge carriers, excitons, charge transfer complexes, etc.) that play key roles in the photoelectric conversion mechanism. These techniques include transient absorption spectroscopy (TAS) [16–18] and photoluminescence lifetime spectroscopy (PLS) [19–21] using ultraviolet (UV)–visible (VIS)–infrared (IR) light pulses [the frequency is sub-PHz (10^15^ s^−1^)], time-domain terahertz (10^12^ s^−1^) spectroscopy (TD-THz) [22–24], and time-resolved microwave conductivity (TRMC) using gigahertz (10^9^ s^−1^, GHz) microwaves [25–30]. Electron paramagnetic resonance (or electron spin resonance) [31, 32] and nuclear magnetic resonance [33, 34] using GHz and MHz (10^6^ s^−1^) EMWs, respectively, in the presence of an external magnetic field provide partial information linked to the dynamic behaviors of molecules and short-lived species. A pulsed X-ray [its frequency is EHz (10^18^ s^−1^)] from a free electron laser is currently used to study ultrafast structural dynamics [35, 36]. Owing to the high transmittances of EMWs in a free space (vacuum, air, and gas), these spectroscopies allow electrodeless measurements, in contrast to normal contact-mode measurements such as device characterization and MHz impedance spectroscopy that require metal electrodes to apply voltage and/or extract charges from a semiconductor. Having considered the photon energies of EMWs and their photophysical interaction with matter, TAS and PLS using UV–VIS–IR light reveal rich insight into the electronic transitions and vibrational motion of chemical bonds specific to transient species, while GHz and THz spectroscopies provide insight into the local oscillation motion of charges and rotational motion of dipoles. TAS and TD-THz with femtosecond time resolution require an expensive femtosecond laser system, an expertized setup of optics, and long-term averaging to obtain a very small signal, whereas TRMC using GHz EMW utilizes a relatively inexpensive excitation source (e.g., a nanosecond laser), an easy circuit setup, and a high-sensitivity resonant cavity. In addition, scattering of EMW, which is significant in UV–VIS light incident on a rough surface sample, is negligible in the GHz region; thus, TRMC is compatible with various forms of samples, including films, powders, liquids, and gases, but not with highly conductive materials (metals and highly doped semiconductors) and dipole-rich condensed matter (e.g., water).

Based on the unique interaction of GHz EMW with matter and its instrumental advantages, the author has developed a TRMC system for the evaluation of photo energy conversion materials. This review describes the fundamentals of GHz spectroscopy and its application to a study on material science.

## Basic of TRMC

**Fig. 2:**
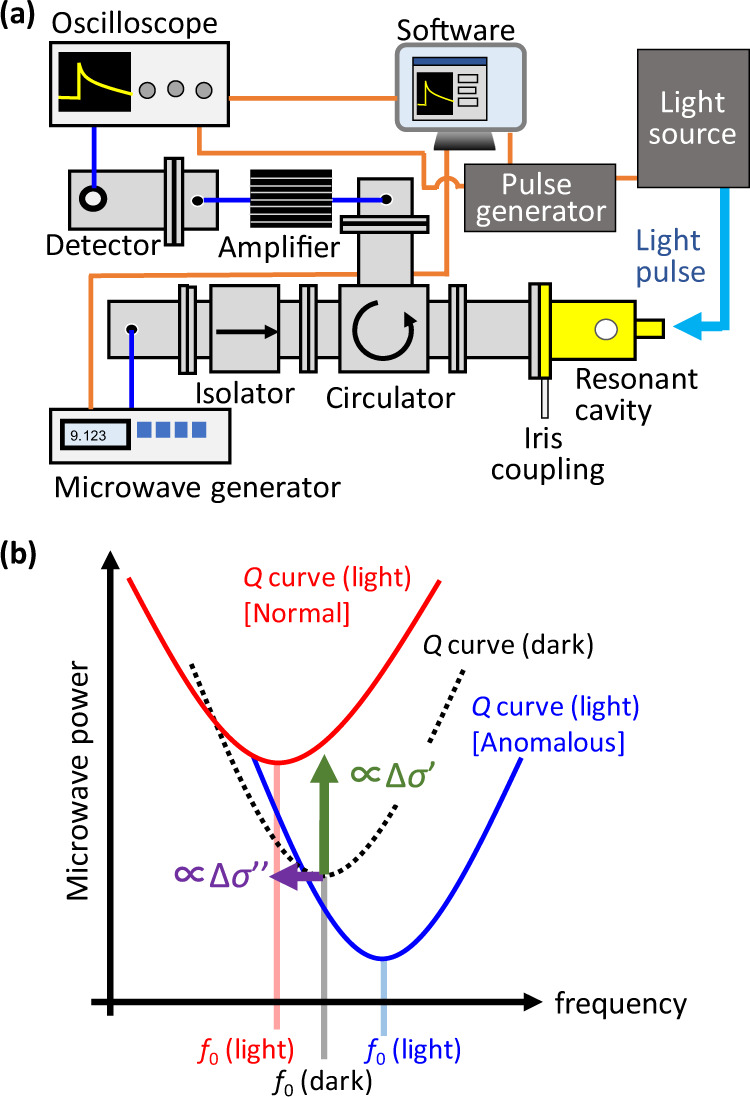
**a** Schematic of a TRMC system. The component in gold color is a resonant cavity. The pipes attached to the cavity are used to introduce excitation light and a sample while confining the microwave inside. **b** Schematic of the change in the *Q* curve at an undercoupling condition (see Eq. (3)). The dotted line represents the *Q* curve in darkness, the red solid curve on the upper side represents the normal transient *Q* curve upon light exposure [40], and the blue solid curve on the lower side represents an anomalous transient *Q* curve upon light exposure [48]. Shifts in the vertical (microwave power) and horizontal (resonant frequency) directions correspond to the real (Δ*σ*′) and imaginary (Δ*σ*″) components of photoconductivity, respectively (usually |Δ*σ*′| >> |Δ*σ*″|)

Figure 2a shows a schematic of a TRMC system, where the rectangular component with gold (yellow) color represents a resonant cavity (generally a transverse electric mode of TE_102_) containing a sample. The microwave circuit consists of a resonant cavity, a microwave source (a Gunn oscillator or a microwave generator), an isolator, (an attenuator), a circulator, a microwave amplifier, and a microwave detector. For a flash-photolysis (FP)-TRMC measurement using a UV–VIS–IR light pulse as the excitation, the transient photoconductivity (Δ*σ*) induced by photogenerated charge carriers is quantified by [37, 38]

1 $${\Delta} \sigma = \frac{1}{A}\frac{{{\Delta} P_{\mathrm{r}}}}{{P_{\mathrm{r}}}},$$

2 $$A = \frac{{ \mp Q\left( {1/\sqrt {R_0} \pm 1} \right)}}{{\pi f_0\varepsilon _0\varepsilon _{\mathrm{r}}}},$$

where *A*, Δ*P*_r_, and *P*_r_ are the sensitivity factor, the small change in the microwave power reflected from the resonant cavity upon photoexcitation, and the microwave power reflected from the resonant cavity in the dark, respectively. *R*_0_ is the ratio of the reflected (*P*_r_) and incident (*P*_i_) microwave power (*R*_0_ = *P*_r_/*P*_i_), and *ε*_r_ and *ε*_0_ are the relative dielectric constant inside the cavity and the dielectric constant in vacuum, respectively. *f*_0_ is the resonant frequency of the resonant cavity. *Q* is the *Q* (quality) value of the resonant cavity associated with the number of microwave reflections and the load in the cavity, which relates to the sensitivity of the measurement. Experimentally, *Q* is estimated by *f*_0_/Δ*f*_1/2_, where Δ*f*_1/2_ is the full bandwidth at half maximum of the reflected microwave power profile near *f*_0_. The polarity (±) of *A* corresponds to the coupling condition (+: undercoupling or −: overcoupling) of the waveguide and cavity, which can be tuned by an iris coupling equipped between the waveguide and the resonant cavity. Since *A* is experimentally determined, a time evolution of Δ*σ* is readily obtained for each measurement. The power of the probing microwave is attenuated to a few to tens of milliwatts so that the electric field of the microwave neither disturbs the motion of charge carriers nor heats the sample (a microwave oven is a few hundred watts). As a trade-off of a highly sensitive resonant cavity, the measurement frequency is fixed for each microwave circuit, and the time resolution deteriorates to a few to one hundred nanoseconds, approximately given by *Q*/*f*_0_.

Analogous to the rheology of viscoelastic materials and the complex refractive index of optics, the Δ*σ* obtained in GHz and THz spectroscopies is in a complex form composed of real (Δ*σ*′) and imaginary (Δ*σ*″) components at a given frequency (*f*). Δ*σ*′ and Δ*σ*″ correspond to the small changes in the imaginary (Δ*ε*″) and real (Δ*ε*′) parts of complex permittivity, respectively, through Δ*σ*′ = *ε*_0_*ω*Δ*ε*″ and Δ*σ*″ = −*ε*_0_*ω*Δ*ε*′, where *ω* is the angular frequency (=2π*f*) [39, 40]. Transient charge carriers generally cause an increase in a complex *ε* (Δ*ε*′ > 0 and Δ*ε*″ > 0), leading to a positive Δ*σ*′ and negative Δ*σ*″ [40, 41]. The frequency dispersion of Δ*σ*(*f*) in the THz region is measured by TD-THz and often analyzed by a Drude–Smith model [42], whereas that in the GHz region measured by TRMC is not at one time due to the fixed resonant frequency. The formulas in Eqs. (1) and (2) correspond to the small change in the real part (Δ*σ*′) associated with the dielectric loss, while the imaginary part (Δ*σ*″) appears as the transient change in the resonant frequency, as illustrated in Fig. 2b. The first perturbation theory of a resonant cavity leads to [39]

3 $${\Delta} \left( {\frac{1}{Q}} \right) - i\frac{{2{\Delta} \omega _0}}{{\omega _0}} = \frac{F}{{\varepsilon _0\omega _0}}\left( {{\Delta} \sigma ^\prime - i{\Delta} \sigma ^{\prime\prime}} \right),$$

where Δ*ω*_0_ and *F* are the shift in the resonant angular frequency and the geometry factor considering the spatial overlap between the electric field and the sample in the resonant cavity. The second perturbation theory gives basically the same expression [43]. When Δ*σ*′ is small, Δ(1/*Q*) in Eq. (3) is approximated to be proportional to Δ*P*_r_/*P*_r_, as given by Eq. (1), which is confirmed by a numerical calculation [44].

The real part of photoconductivity (Δ*σ*′) is converted to the product of the quantum yield of charge carrier generation (*φ*) and the sum of the charge carrier mobilities (Σ*μ*) (=*μ*_h_ + *μ*_e_, *μ*_h_: hole mobility, *μ*_e_: electron mobility) by

4 $$\varphi {\sum} {\mu} = \frac{1}{{e \cdot I_0 \cdot F_{{\mathrm{Light}}}}} \cdot {\Delta} \sigma ,$$

where *e*, *I*_0_, and *F*_Light_ are the unit charge of a single electron, the excitation photon density of the laser (photons cm^−2^) and the correction factor (cm^−1^). *F*_Light_ is calculated by homemade software that considers the sample geometry, the photoabsorption of the sample at the excitation wavelength, the laser spot and position, and the spatial overlap of the laser and electric field in a cavity calculated by an electrostatic simulation [44, 45]. Note that *φ* and Σ*μ* are time-dependent when the disappearance of charges through recombination and trapping and charge relaxation are involved, respectively.

**Fig. 3:**
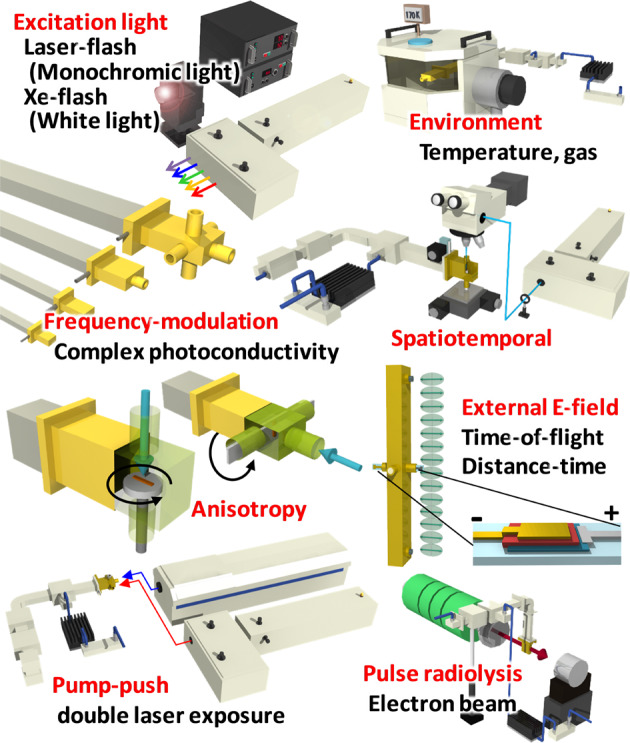
Drawing of TRMC options that the author has developed

Considering the nanosecond time response of a resonant cavity, a nanosecond laser (typically <10 ns in pulse width) such as a Nd:YAG (yttrium aluminum garnet) [the fundamental light: 1064 nm, the second harmonic generation: 532 nm, the third harmonic generation (THG): 355 nm, the fourth harmonic generation: 266 nm] and an optical parametric oscillator (e.g., 300–1000 nm) seeded by the THG of a Nd:YAG are usually used for excitation. These lasers emit monochromic light, while the performance of a solar cell is an overall electric output under exposure to broad-spectrum sunlight. As shown in Fig. 3, the author developed an excitation light source that utilizes a white light pulse from an in-house Xe-flash lamp, which is suitable for the evaluation of solar cell materials (vide infra) [46]. To gain mechanistic insight into charge transport, temperature-controlled experiments are performed in a vacuum chamber (a low temperature of ~77 K to a high temperature of ~400 K), together with the change in gas (air, nitrogen, oxygen, etc.) in a closed cavity [47]. The most common microwave frequency of TRMC is X band (~9 GHz), while other frequencies such as the K_u_ band (~15.4 GHz), K band (~22.9 GHz), and Q or K_a_ band (~33.2 GHz) are available [40, 47, 48]. Moreover, measuring the photoconductivity transients at modulated frequencies near *f*_0_ and reconstructing the transient *Q* curves by means of in-house software enable the separation of Δ*σ*′ and Δ*σ*″ on the basis of Eq. (3) [40, 41]. This evaluation can reveal the depth and density of charge traps [40] and anomalous dielectric behavior in organic–inorganic perovskites [48] (Fig. 2b). Although Δ*σ* and *φ*Σ*μ* arise from the local (nanometer scale) motion of charges and dipoles, these values are averaged over the exposed light spot (a few millimeters). Accordingly, a spatiotemporal option was developed by embedding an optical microscope that focuses the laser spot to ~45 μm in size [49]. Although the spatial resolution is too low to visualize the crystal grains of perovskite (<1 μm), the measurement revealed the spatial inhomogeneity of Δ*σ* in halogen-mixed perovskite [MAPb(I_1–*x*_Br_*x*_)_3_, MA: methylammonium cation] along the distance from the center of spin coating. In addition to an increased sensitivity of the resonant cavity, the direction of the electric field inside is fixed. Based on this property, anisotropic photoconductivity and mobility along various sample directions (parallel and perpendicular to a film and the *xyz* axes of a crystal) are easily evaluated with a high angular resolution [50–62].

As a drawback of high-sensitivity resonant cavities, solar cell devices are incompatible with TRMC measurements because of the absorption and/or reflection of microwaves by the gold, aluminum, silver, or indium tin oxide electrodes in the devices. Nonetheless, charge carrier dynamics under not only open-circuit conditions (no electrodes) but also short-circuit conditions (charge extraction by electrodes) are of interest. The author developed a high-mode, long-shaped cavity (TE_10*m*_, *m* = 14) that enables TRMC measurements of an OPV device by applying an external electric field [63]. By using this system, time-of-flight (TOF) and TRMC measurements were simultaneously conducted, which elucidated hole relaxation in spatial (nm–μm) and temporal (μs) aspects (vide infra). A pump–push measurement using a UV pump pulse and an IR push pulse from two laser systems has been developed and used to investigate a charge trap and its relaxation [48, 64]. Pulse radiolysis using a pulsed electron beam from a high-energy accelerator is an effective method for quantitative, homogeneous injection of charges in a solution and block sample [28, 37], whereas light injection has an uncertainty of *φ* that depends on the sample, wavelength, intensity, and time range. Other notable TRMC options include field-induced TRMC [65], TRMC under pressure in a hydrostatic medium (<0.15 GPa) [66], steady-state microwave conductivity using chopped light and a lock-in amplifier [67, 68], and spatiotemporal imaging of microwave conductivity [69], highlighting the diversity and versatility of GHz spectroscopies.

## Photoelectric conversion materials: OPVs, perovskite, and photocatalysts

Owing to the electrodeless measurement of the local motions of charge carriers, laser-flash TRMC has been extensively used in liquid crystalline materials [55, 70–72], self-assembled nanostructures [50, 60, 73–80], microcrystals [52, 56, 81–84], and liquids/gels [85–88], which are often associated with difficulties in preparing high-quality films suitable for device characterization, such as a field-effect transistor (FET). Although one can prepare a smooth film of a conjugated polymer, the *φ* value of its pristine film is very small (10^−5^–10^−3^) and unable to be determined solely by TRMC measurements. To address this issue, the author incorporated perylenebisimide (PBI) into a poly(3-hexylthiophene) (P3HT) film, which acts both as an electron acceptor to increase *φ* and as a spectroscopic probe to determine *φ* [89, 90], because the PBI radical anion has a characteristic photoabsorption and a large extinction coefficient at ~720 nm [91]. From the simultaneous measurements of TRMC (*φ*Σ*μ*) and TAS (*φ*), the TRMC hole mobilities (Σ*μ* ≈ *μ*_h_) in regioregular and regiorandom P3HT films were determined to be ~0.1 and 0.006 cm^2^ V^−1^ s^−1^, respectively [89]. Notably, the former was considerably decreased when a large amount of PBI (e.g., >41 wt%) was mixed in due to the disturbed lamellar formation of P3HT.

**Fig. 4:**
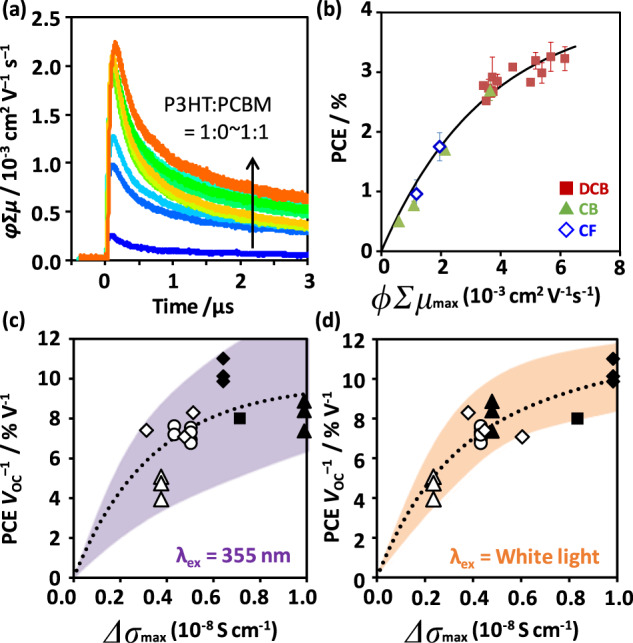
TRMC evaluations of OPV films. **a** *φ*Σμ transients of P3HT:PCBM with changing blend ratio (1:0 as the blue line to 1:1 as the orange line). The excitation wavelength (*λ*_ex_) was 355 nm. **b** PCE vs. *φ*Σ*μ*_max_ of P3HT:PCBM (1:1) with different solvents (DCB, CB, CF) and thermal annealing temperatures. *λ*_ex_ = 355 nm. The solid line is a least-mean-square fitted inverse exponential function. **c** PCE *V*_OC_^–1^ vs. Δ*σ*_max_ of NBPs:PCBM (*λ*_ex_ = 355 nm). The circle, triangle, square, and diamond correspond to NBPs:PCDTBT, PCPDTBT, PBDTTPD, and PBDTTT-CF, respectively. The open and closed symbols are without additive and with 1,8-diiodooctane (DIO), respectively. **d** PCE *V*_OC_^–1^ vs. Δ*σ*_max_ of NBPs:PCBM (*λ*_ex_ = white light pulse). **a**, **b** and **c**, **d** are reproduced from [92] (Copyright: 2011 Wiley-VCH) and [46] (Copyright: 2012 American Chemical Society), respectively, with permission

A mixture of a donor and an acceptor is similar to the BHJ framework of OPVs, while soluble fullerenes ([6,6]-phenyl-C_61_-butyric acid methyl ester: PCBM and [6,6]-phenyl-C_71_-butyric acid methyl ester: PC_71_BM) are commonly used as n-type semiconductors. The author next investigated the correlation of TRMC signals with OPV device performance. Figure 4a shows the *φ*Σ*μ* transients of regioregular P3HT films blended with PCBM upon exposure to 355 nm light [92]. With increasing PCBM content, the maximum *φ*Σ*μ* (*φ*Σ*μ*_max_) at the time resolution limit increased considerably and reached 2.3 × 10^−3^ cm^2^ V^−1^ s^−1^ at P3HT:PCBM = 1:1. A further increase in PCBM led to decreases in the *φ*Σ*μ*_max_ and lifetimes of decays. Notably, the product of *φ*Σ*μ*_max_ and the half lifetime (*τ*_1/2_) indicated the distinct maximum at p:n = 1:1, which is the optimal blend ratio in P3HT:PCBM OPVs suggesting the importance of *μτ*_1/2_ in the device performance. The large *φ* and Σμ are related to the high density of photogenerated charge carriers and the high yields of charge separation/transport, respectively, while the large *τ*_1/2_ results in efficient charge transport and collection with minimal loss. By separately characterizing *φ* and Σ*μ* in various narrow-bandgap polymers (NBPs) and PCBM blends, Σ*μ* associated with the crystallinity and crystallite size of the polymer domain was suggested to have a positive impact on charge separation [93]. A large local mobility is presumably a key factor that facilitates charge separation to break the strong Coulombic potential of intermolecular excitons [21, 94, 95].

TRMC evaluations are useful for screening not only p:n ratios but also process conditions. Figure 4b shows the plot of PCE vs. *φ*Σ*μ*_max_ of different solvents (DCB: ortho-dichlorobenzene, CB: chlorobenzene, and CF: chloroform) and thermal annealing (no annealing and annealing at 140–160 °C) found in P3HT:PCBM = 1:1 films (the excitation is 355 nm) [92]. A pronounced, sublinear correlation was observed between PCE and *φ*Σ*μ*_max_, demonstrating that TRMC allows rapid optimization of the process conditions without delicate fabrication of OPV devices. The addition of a Pd catalyst into P3HT:PCBM devices caused a significant drop in PCE (approximately half at 5 wt% Pd), whereas its effect on *φ*Σμ_max_ was marginal (~10% decrease), highlighting the tolerance of TRMC measurements against impurity and degradation (no change for 1 week of storage in air).

Despite the good correlation between TRMC and the OPV performances found in P3HT:PCBM, that in other NBP:PCBM films, deteriorated, as shown in Fig. 4c. This is because NBPs have variations in their photoabsorption, optimal film thickness, photocurrent generation, charge carrier lifetimes, etc. Scanning the excitation wavelength from UV to VIS to IR and integrating the results by considering the sunlight spectrum is technically possible but eliminates rapid measurement. Accordingly, the author developed a Xe-flash TRMC system using a white light pulse for excitation [46]. Good spectral matching of the white light pulses was confirmed by comparison with sunlight. The pulse width was tuned to ~10 μs by considering the intensity of the TRMC signal and a typical transit time of charge carrier collection [96]. The decrease in time resolution (~10 μs for Xe-flash TRMC and ~40 ns for laser-flash TRMC) is rather useful because the photoconductivity maximum (Δ*σ*_max_) of Xe-flash TRMC convolutes both *φ*Σ*μ*_max_ and the lifetime (i.e., *μτ*_1/2_ value), and the light intensity of the white light pulse is much closer to that of sunlight than to that of a laser, allowing a direct comparison of this value (Δ*σ*_max_). The correlation of PCE *V*_OC_^−1^ with the Δ*σ*_max_ values observed in NBPs (Fig. 4d) and p:n ratios (not shown in this article) is therefore improved, making Xe-flash TRMC versatile for material and process screening of OPV.

**Fig. 5:**
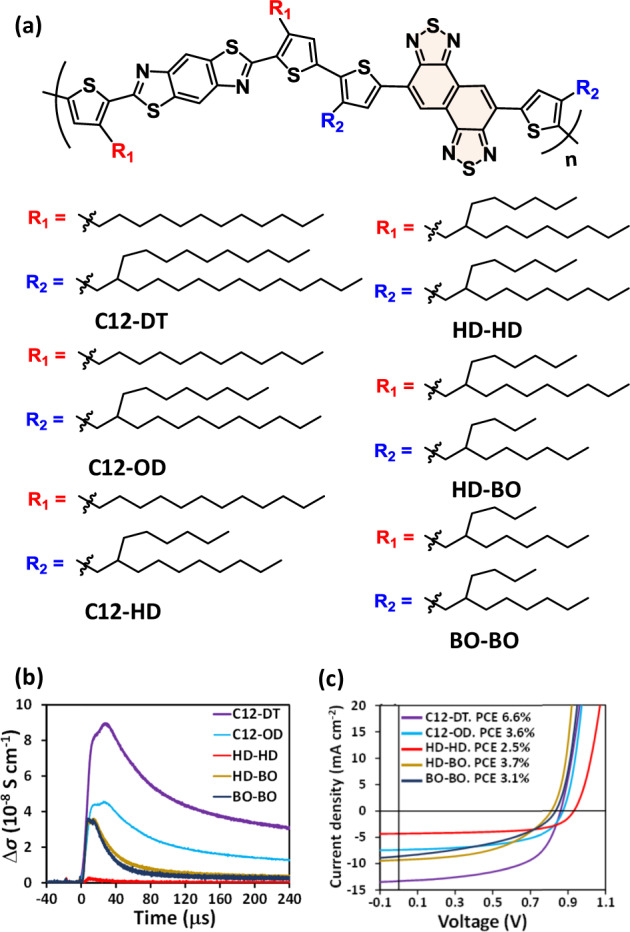
**a** Chemical structures of PBBTzNTz polymers. **b** Δ*σ* transients of PBBTzNTz:PCBM films at their optimal blend ratios measured by Xe-flash TRMC (*λ*_ex_ = white light pulse). Each decay corresponds to the respective alkyl chain. **c** Current density vs. voltage curves of the PBBTzNTz:PCBM devices. Their PCE values are appended. Reproduced from [100] with permission (Copyright: 2017 American Chemical Society)

By using Xe- and laser-flash TRMC, new conjugated polymers for OPVs were explored. Benzobisthiazole (BBTz), shown in the left component of the polymer in Fig. 5a, is a weak electron acceptor but could act as a weak electron donor against strong electron acceptors. First, the author synthesized three random copolymers of BBTz coupled with benzothiadiazole (BT) or thienopyrroledione (TPD) as the acceptor and carbazole as the donor [97]. Although the solubilities of these polymers are insufficient due to the nonoptimized alkyl chains, TRMC evaluations of their PCBM blends were successfully performed, showing the largest Δ*σ*_max_ for BBTz–BT. Based on this feedback, an alternating copolymer of BBTz–BT with branched long alkyl chains (identical to C12-DT in Fig. 5a) was synthesized and applied to OPV characterization. As a result, a high PCE of 6.5% was obtained, which established evaluation-oriented material exploration [98]. Second, the BT unit was modified by fluorinated BT [99], pyridine thiadiazole [99], and naphthobisthiadiazole (NTz) [100]. In the case of NTz, six polymers with different combinations of alkyl chains were synthesized to examine their effects (Fig. 5a). TRMC screening of p:n ratios revealed that the optimal ratio is ~1:2. Figure 5b displays the Δ*σ* transients of different alkyl chains at their optimal p:n blend ratios. The Δ*σ* of the purple-colored profile (C12-DT) is the largest, followed by the white blue (C12-OD), dark blue (BO-BO) = dark yellow (HD-BO), and red (HD-HD) profiles. The PCE values of these OPVs were exactly the same as those determined by the TRMC evaluations (Fig. 5c), where the maximum PCE of C12-DT was 6.6%. The PCE variations despite the identical polymer backbone was attributed to their BHJ morphologies. C12-DT exhibited microfiber structures of polymer crystallites on the atomic force microscopy images, whereas the microfibers disappeared in BO-BO, and sphere-shaped aggregates were observed in the lowest-performing HD-HD. To date, no general rule on the design of side alkyl chains has been established; thus, researchers have to synthesize plausible combinations of alkyl chains for each backbone.

**Fig. 6:**
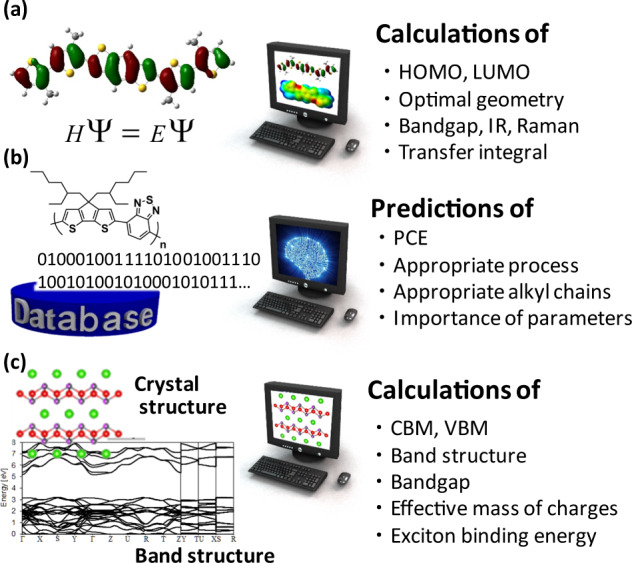
Conceptual images of computer-aided evaluations and predictions. **a** Conventional quantum chemical calculations such as DFT. **b** ML-based prediction of OPV materials. **c** Quantum chemical calculations of inorganic semiconductors in band theory

Despite rapid screening of OPV materials by TRMC, the experimental measurements require realistic materials. Furthermore, a calculation-driven exploration of materials has become increasingly attractive over the past three decades [101–104] due to continuous improvements in computer hardware, software, and quantum chemical theory. Quantum chemical calculations such as density functional theory (DFT) together with Marcus electron transfer theory and molecular dynamics simulations enable a virtual evaluation of subjected molecules, which quantifies fundamental properties such as HOMO, LUMO, *E*_g_, a transfer integral, and mesoscale molecular conformations (Fig. 6a) [105]. However, these calculations are computationally expensive to obtain high-accuracy outputs. Irrespective of the high-cost calculations, these properties do not always correlate with the OPV performance because many multidimensional factors (solubility, intermolecular interactions, miscibility, solvent, etc.) affect the BHJ morphology and interfacial energetics in a complex manner. Along these lines, data science—a counterpart of computer science and empowered by ever-growing artificial intelligence—is rising to prominence as extremely high-throughput material screening. Accordingly, materials informatics (MI) utilizing machine learning (ML) technology has been deployed for inorganic thermoelectric materials [106, 107], inorganic semiconductors [108–110], and molecular drugs [111, 112]. For these materials, there is a relatively close relationship between the calculated values and material function because the electronic properties of a periodic framework of inorganic compounds or a single molecule exhibit a direct influence on their functions. Although MI-driven molecular exploration in organic electronics is challenging [104, 113–115], attempts have been made in FET molecules [116, 117], thermally activated delayed fluorescence molecules [118, 119], and OPV materials [120–123].

The author extended his research on experimental screening by TRMC to virtual screening by ML (Fig. 6b) [121]. An ML study needs a large database; however, such a database specific to OPV is unavailable, in contrast to well-compiled inorganic databases. Accordingly, OPV data (~1200 entries from ~500 papers) were manually collected from the literature, which includes the step-by-step pickup of OPV parameters (polymer:PCBM/PC_71_BM) and physical properties (*M*_n_, *M*_w_, PDI, *E*_g_, HOMO, LUMO) and conversion of chemical images to digital structures [a ChemDraw format and simplified molecular input line entry system (SMILES)]. The SMILES was then converted to a Boolean series by checking the 1024 digital numbers of an extended connectivity fingerprint (ECFP6) key of each chemical structure. ECFP6 is a high-resolution fingerprint that considers the neighboring connectivity of atoms and is calculated by the RDKit tool of R Studio (R language) or Anaconda (Python) software. ML with the random forest (RF) algorithm that uses multiple decision trees and averages all these outputs yielded a Pearson’s correlation coefficient (*r*) of 0.62. Although this *r* value is still unsatisfactory (1 for a perfect positive correlation), this ML allows rapid material screening (<1 s for 1000 structures) and a prediction of appropriate alkyl chains for each polymer backbone, which is impossible in conventional DFT calculations. In this ML, the chemical structures (ECFP6 keys) and physical properties (*M*_w_, *E*_g_, etc.) of polymers were used as the input (explanatory variables), and PCE was set as the output (an objective variable), where the importance of the RF algorithm revealed that the most important explanatory variables were *M*_w_, *E*_g_, and some of the fingerprints. The choice of algorithm is also crucial to prediction accuracy; for example, an artificial neural network (ANN)—often used in image and voice recognition—resulted in a very low *r* of 0.37. This is possibly because RF is tolerant of noise data (incorrect or exceptional data) and leads to high accuracy even in a small dataset, whereas ANN requires a large dataset (>tens of thousands entries) with minimal noise data included. The ML study of polymer:fullerene acceptor (FA) was further evolved to polymer:nonfullerene (NFA) that recently demonstrated a remarkable improvement in PCE of up to ~18% [124–127]. The RF ML model of polymer:NFA (~500 entries) constructed in the same way as that of polymer:FA exhibited an improved *r* of 0.85 [128, 129]. This improvement is probably due to the almost doubled inputs (polymer and NFA data) and improved data accuracy reported in recent years. Surprisingly, this model showed good agreement between the predicted PCE (11.2%) and the experimental PCE (11.0%, average: 10.6 ± 0.2%) for a new polymer and NFA [128]. Furthermore, the predicted appropriate alkyl chains (*n*-octyl or 2-ethylhexyl) and process conditions (solvent, additive, thermal annealing, and p:n blend ratio) were consistent with the experiments. These works open up an attractive avenue to the effective exploration of complex photoelectric systems from experimental and virtual spaces, which may be increasingly important in the recent paradigm shift caused by coronavirus outbreaks.

Vigorous investigations on blooming perovskite solar cells (PSCs) have been conducted by using TRMC techniques, including charge carrier dynamics [47, 130–134], charge transfer to hole and electron transfer layers assisted by data science analysis [135], and the development of lead-free materials (Sn [48, 136, 137] and Bi/Sb [138–140]). The revealed TRMC mobilities (10–100 cm^2^ V^−1^ s^−1^) of lead halide perovskites [47, 134, 135] are much larger than those of organic semiconductors (0.1–1 cm^2^ V^−1^ s^−1^) and account for the high PCE of PSCs. A unique GHz dielectric behavior ascribed to the rotational motion of organic cations is also notable [48]. In addition to these photoelectric conversion systems (OPV and PSC), inorganic photocatalysts that generate oxygen and/or hydrogen from water and sunlight energy are materials of interest. Based on knowledge obtained in the GHz dielectric behavior of PSC, the author directly probed the interplay of charge trap and elemental stoichiometry (Sr/Ti) of SrTiO_3_, a paraelectric perovskite used as the hydrogen-generating photocatalyst [141]. Bi_4_TaO_8_Cl and PbBiO_2_Cl photocatalysts [142] showed good correlation of *φ*Σ*μ*_max_ × *τ*_1/2_ with their oxygen evolution rates, where *φ*Σ*μ*_max_ increased monotonically with the calcination temperature due to the improved crystallinity evaluated by XRD, while *τ*_1/2_ decreased in line with the halogen defect found by X-ray photoelectron spectroscopy [143].

The complexity of photochemical reactions is significant in photocatalysts, as they involve photoabsorption, charge separation and transport, and oxidative/reductive reactions on the solid/liquid interface facilitated by cocatalysts. Nonetheless, one of the most primitive parameters of inorganic semiconductors in band theory is the effective mass of charge (m*), which is related to the mobility (*μ*) as follows (Fig. 6c) [144]:

5 $$\mu = \frac{{e\tau }}{{m \ast }},$$

where *τ* is the collision (scattering, relaxation) time of the charge carrier. m* is calculated from the double partial differentials of the curvature in the band diagram given by [144]

6 $$m \ast = \hbar ^2\left( {\frac{{\partial ^2E\left( k \right)}}{{\partial k^2}}} \right)^{ - 1},$$

where ℏ is the reduced Planck constant (the Dirac constant), *E*(*k*) is the energy profile corresponding to the valence band maxima for a hole and conduction band minimum for an electron, and *k* is the wavenumber. In band theory, the exciton binding energy (*E*_bind_) is estimated by the Rydberg equation for a hydrogen-like atom expressed by [144]

7 $$E_{{\mathrm{bind}}} = \frac{{e^4}}{{2\hbar ^2\varepsilon ^2}}\frac{{m_{\mathrm{e}}\!\! \ast m_{\mathrm{h}} \ast }}{{m_{\mathrm{e}}\!\! \ast + m_{\mathrm{h}} \ast }},$$

where *m*_h_* and *m*_e_* are the effective masses of a hole and electron, respectively. From Eqs. (5–7), one can readily expect that a small effective mass of charge leads to a small *E*_bind_ and a large *μ* that merit efficient charge separation, charge transport, and consequently the optoelectronic output. Furthermore, the relationship among the calculated m*, experimental *μ*, and photocatalytic activity has remained elusive because of the abovementioned complexity of photocatalytic systems. However, this relationship was revealed in PbBiO_2_Cl and SrBiO_2_Cl photocatalysts, where the mixing of Sr and Pb formed a solid solution at arbitrary ratios [62]. Both *m*_h_* and *m*_e_* calculated by DFT increased with increasing Pb content, which corresponded to decreases in *φ*Σ*μ*_max_ (the change in *τ*_1/2_ is insignificant) and the oxygen evolution rate. Such a fundamental correlation was observed for the first time, owing to the continuous replacement of Sr by Pb and insignificant change in the particle morphology and crystal polymorph. Since m* is obtained by quantum chemical calculations or predicted by ML, this work encourages efficient exploration of potential semiconducting photocatalysts based on transient spectroscopies and computed parameters.

## Beyond ML: a regression to experiments

**Fig. 7:**
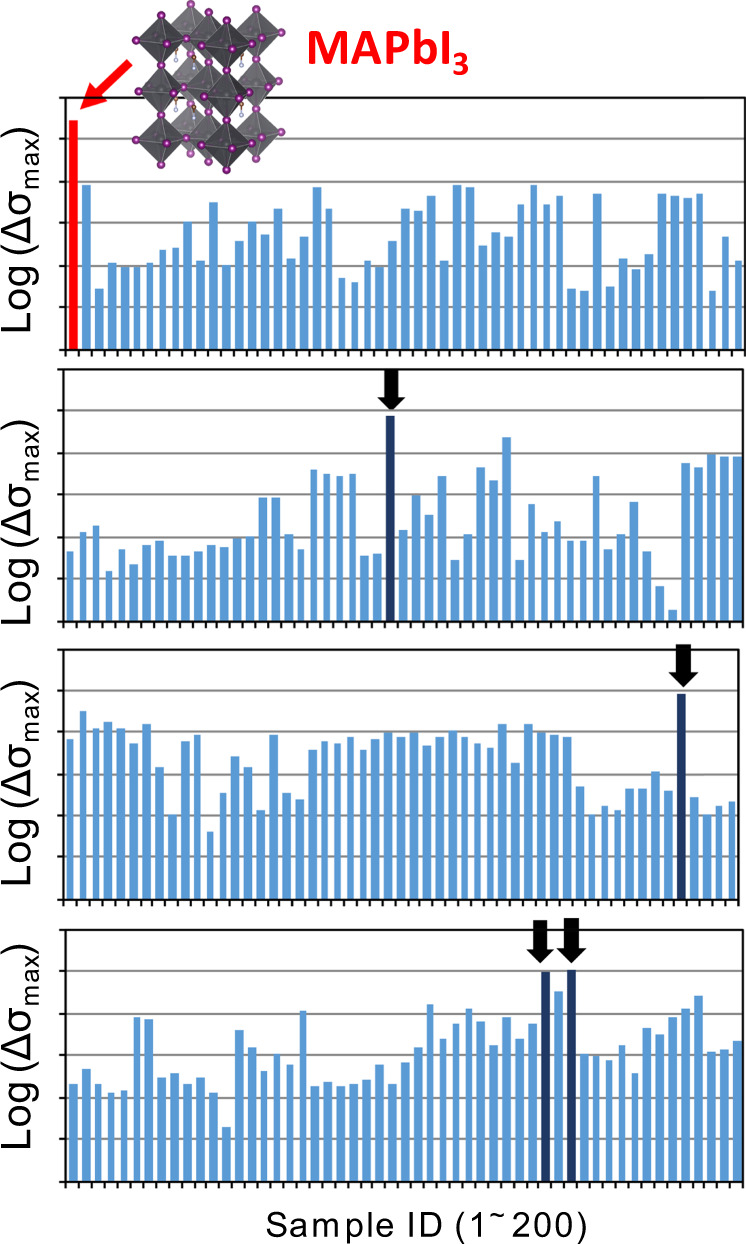
Rapid experimental screening of inorganic and organic–inorganic hybrid semiconductors by using Xe-flash TRMC. The vertical axis is the common logarithmic plot of Δ*σ*_max_ (arbitrary unit). The red bar represents the Δ*σ*_max_ of the highly efficient perovskite of MAPbI_3_ along with its structure. The dark blue bar with arrows represents the materials that show relatively high Δ*σ*_max_ values

ML allows the prediction of complex systems with acceptable accuracy by extremely high-speed screening over millions of candidates, which is impossible by human brains [101–104]. However, ML predictions have uncertainty and are biased on the learned data, including success and failure; thus, the author believes that experiences, inspirations, and serendipity of researchers are undoubtedly necessary for the substantial revolutions and breakthroughs that science has sometimes encountered in its history [145, 146]. Rapid TRMC screening of photoelectric materials is therefore a sound method to find a plausible candidate that has not yet been thoroughly explored. Figure 7 shows the logarithmic Δ*σ*_max_ values of over 200 semiconductors (inorganic and organic/inorganic hybrids) measured by our Xe-flash TRMC. These values were sequentially measured when the materials were synthesized in the laboratory, bought from companies, and supplied from collaborators. The highly efficient organic–inorganic perovskite of MAPbI_3_ (red bar in the figure) exhibited a very high Δ*σ*_max_ relevant to the high PCE of PSC devices. Most of the Δ*σ*_max_ values of other semiconductors were, however, two to five orders of magnitude smaller than that of MAPbI_3_. Notably, four materials indicated by the dark blue arrows in the figure exhibited high Δ*σ*_max_ values, although they were still one order of magnitude smaller than that of MAPbI_3_. One such material is bismuth sulfide (Bi_2_S_3_) powder, which is a two-dimensional metal chalcogenide with a semiconductive nature [147, 148] but is mostly unexplored because of problems in preparing a high-quality film.

Despite the insoluble nature of Bi_2_S_3_ in organic solvents, film fabrication is reportedly possible through colloidal nanocrystal suspension (NC) [149], chemical bath deposition [150], successive ionic layer adsorption reaction [151], spray pyrolysis [152], and a precursor of bismuth triethyldithiocarbonate (xanthate) (Bi(EtX)_3_) [153]. However, these methods result in a trade-off between the electronic and morphological qualities, i.e., a film prepared by NC yields a smooth surface but a low crystallinity and a small size [149]; a film prepared from Bi(EtX)_3_ yields high crystalline Bi_2_S_3_ but contains rod-shaped morphologies [153]. Accordingly, the author developed a novel film preparation method named chemically assisted spin coating and crystallization (CASC) that solves this issue by separating the crystal nucleation (spin coating of a precursor solution) and growth (under diluted H_2_S gas) steps [154]. Many process conditions (precursors, solvents, time and temperature of thermal annealing, flow of H_2_S gas) were screened by monitoring the Δ*σ*_max_ values of Xe-flash TRMC as a figure of merit. As a result, a high-quality Bi_2_S_3_ film was successfully realized by the CASC method, which satisfies both a high electronic quality (Hall effect electron mobility ∼7 cm^2^ V^−1^ s^−1^) and a low surface roughness (1.7 nm) with a large grain size (<400 nm). A photoresistor (photodetector) fabricated with this film exhibited a high detectivity of 1.7 × 10^11^ Jones (cm Hz^1/2^ W^−1^) comparable to that of MAPbI_3_, along with a long-term stability (>3 months). This work again exemplifies an effective research concept of evaluation-oriented exploration of photo energy conversion systems.

**Fig. 8:**
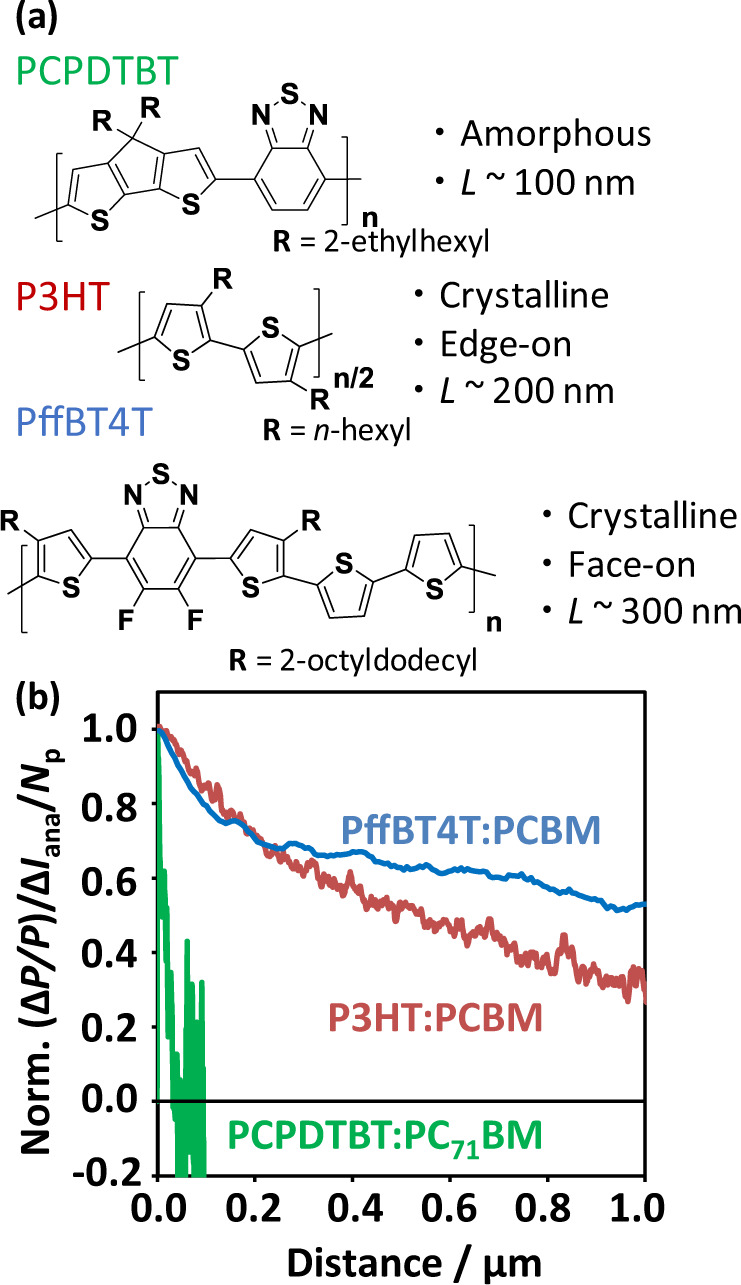
**a** Chemical structures of OPV polymers used in the TOF–TRMC measurements. Their crystalline properties are appended with their optimal film thicknesses (*L*) of OPV devices. **b** Normalized TRMC decay (Δ*P*/*P*) divided by analyzed photocurrent decay (Δ*I*_ana_) and analytical curves considering bulk recombination and trap (*N*_p_). Each curve shows the hole relaxation profile in the blend films along the travel distance of the hole. Reproduced from [63] with permission (Copyright: 2017 American Chemical Society)

Providing a rationale for the structure–property–function relationship is essential for scientific progress; however, such logical thinking is currently difficult in ML, as it learns only about the relationship between input and output without considering causality (i.e., black box). Moreover, an idea on the development of a new measurement system arises from human thinking (what is a problem, what one wants to know, what is available as an instrumental component, etc.). Therefore, the author regards TRMC as a tool to investigate a scientific basis rather than just to screen materials and processes. To this end, the TOF–TRMC system was developed to investigate the charge carrier dynamics and relaxation on a local scale under an external voltage [63]. The TRMC decays ascribed to holes in NBP:PCBM blend films [poly(cyclopentadithiophene-benzothiadiazole):PCPDTBT, P3HT, and poly(quarterthiophene-difluorinated benzothiadiazole):PffBT4T, as shown in Fig. 8a], were accelerated by applying a voltage, as explained by the accelerated hole collection by the counter negatively biased electrode. By analyzing the transient photocurrent decays, TRMC decays, second-order charge recombination, and first-order trapping under an external electric field, the hole relaxation at the local scale probed by TRMC was deduced as a function of travel distance (Fig. 8b). The relaxation speed is significant in the order of PCPDTBT:PC_71_BM, P3HT:PCBM, and PffBT4T:PCBM, which is consistent with their TOF mobilities, crystalline natures (PCPDTBT: amorphous, P3HT: crystalline with an edge-on orientation, and PffBT4T: crystalline with a face-on orientation), and optimal thicknesses of the OPV devices (PCPDTBT: ~100 nm, P3HT: ~200 nm, and PffBT4T: ~300 nm). Explainability and consistency among various measurements constitute the heart of science that should be pursued in the evaluation-oriented exploration of photo energy conversion systems.

## Concluding remarks

This article reviewed the material exploration and fundamental research of photo energy conversion systems (OPVs, PSCs, photocatalysts, and photodetectors) based on noncontact-mode EMW spectroscopy (TRMC). The unique derivatives of this spectroscopy enable rapid screening of materials and processes suitable for solar cells (Xe-flash TRMC with a white light pulse), investigations of frequency dispersion and charge traps (frequency modulation), charge relaxation in time and distance (TOF–TRMC), and so on. In particular, a research concept combining molecular design and synthesis, TRMC evaluations, and ML-based prediction was demonstrated in the development of π-conjugated polymers for OPVs. The mobility-lifetime product (*φ*Σ*μ*_max_ × *τ*_1/2_) was found to be useful as a figure of merit, which is mostly general in OPV, PSC, and photocatalytic materials. The revealed correlation among m*, *φ*Σ*μ*_max_, and photochemical functions underscores the validity of computer and data science approaches, whereas the inspiration and serendipity of researchers are still pivotal for a discrete revolution of next-generation material science.
